# Effect of prescription isodose line on tissue sparing in linear accelerator‐based stereotactic radiosurgery treating multiple brain metastases using dynamic conformal arcs

**DOI:** 10.1002/acm2.14278

**Published:** 2024-01-17

**Authors:** Yohan A. Walter, Joseph P. Dugas, Bethany L. Broekhoven, Troy D. Jacobs, Muhong Han, Chiachien J. Wang, Hsinshun T. Wu

**Affiliations:** ^1^ Department of Radiation Oncology Willis‐Knighton Cancer Center Shreveport Louisiana USA

**Keywords:** external beam, stereotactic radiosurgery, treatment planning

## Abstract

**Purpose:**

Linear accelerator‐based stereotactic radiosurgery (SRS) has become a mainstay for simultaneous management of multiple intracranial targets. Recent improvements in treatment planning systems (TPS) have enabled treatment of multiple brain metastases using dynamic conformal arcs (DCA) and a single treatment isocenter. However, as the volume of healthy tissue receiving at least 12 Gy (V12) is linked to the probability of developing radionecrosis, balancing target coverage while minimizing V12 is a critical factor affecting SRS plan quality. Current TPS allow users to adjust various parameters influencing plan optimization. The purpose of this work is to quantify the effect of negative margins on V12 for cranial SRS plans managing multiple brain metastases.

**Methods:**

Using the Brainlab Elements v3.0 TPS (Brainlab, Munich, Germany), we calculated V10, V12, V15, monitor units, and conformity index for seventeen SRS plans treating 2–10 metastases on our Elekta Versa HD (Elekta, Stockholm, Sweden) linear accelerator. We compared plans optimized using 70%‐90% prescription isodose lines (IDL) in 5% increments.

**Results:**

Irrespective of the number of treated metastases, optimization at a lower prescription IDL reduced V10, V12, and V15 and increased MU compared to the 90% IDL (*p* < 0.01). However, comparing the 70% and 75% IDL optimizations, there was little difference in tissue sparing. The conformity index showed no consistent trends at different IDLs due to a significant spread in case data.

**Conclusion:**

For our plans treating up to 10 metastases, diminishing returns for tissue sparing at IDLs below 80% paired with increasing treatment MU and dosimetric hot spot made optimization at lower IDLs less favorable. In our clinic, after consulting with a physician, it was determined that optimization at the 80% IDL achieved the best balance of V12, treatment MU, and maximum dose. Clinics implementing LINAC‐based SRS programs may consider using similar evaluations to develop their own clinical protocols.

## 1 INTRODUCTION

Linear accelerators (LINACs) present fast, versatile, and cost‐effective platforms capable of delivering stereotactic radiosurgery (SRS) treatments to multiple cranial targets quickly and simultaneously. Today, the widely used single‐isocenter, multi‐target (SIMT) technique has expedited SRS treatment delivery on linear accelerators. Various works have shown that the SIMT technique can be performed safely, effectively, and quickly, with delivery times of approximately 40 min for up to 10 treated targets.^1^, ^2^, ^3^, ^4^, ^5^

Optimization for the SIMT technique has continuously advanced, with treatment planning systems (TPS) now facilitating delivery using 3‐D dynamic conformal arcs (DCA) or intensity modulation. In dynamic conformal arc delivery, the choice of key planning parameters heavily influences treatment time and tissue sparing. Particularly, current TPS allow users to specify a prescription isodose line (IDL). By selecting an ideal IDL, users can balance monitor unit (MU) efficiency, and therefore treatment delivery time, with optimal tissue sparing.

In a 2011 study, Minniti et al., identified the volume of brain receiving 12 Gy (V12) as a key predictor of radiation‐induced necrosis.^6^ Various recent studies have further confirmed the link between V12 and the probability of radionecrosis in single‐fraction cranial SRS.^7^, ^8^, ^9^ It is, therefore, imperative that SRS plans effectively balance a minimal V12 with adequate target coverage and reasonable treatment time.

When treating single metastases, selection of a lower prescription IDL improves tissue sparing by creating a sharper dose falloff outside the target area, but at the cost of higher treatment MU. However, the overall effect of the prescription IDL on dynamic conformal arc SIMT plans is not as clear‐cut and is dependent on the optimization process. In this work, we present the results of our institutional study comparing tissue sparing for dynamic conformal arc optimization of multi‐target SRS plans using the 70%−90% prescription IDLs.

## 2 METHODS

Using the Brainlab Elements v3.0 TPS (Brainlab, Munich, Germany), we optimized seventeen multi‐metastasis SRS plans using DCA and the SIMT delivery technique. Treatments were planned for MLC‐based delivery on our Elekta VersaHD® linear accelerator equipped with Agility® MLCs (Elekta AB, Stockholm, Sweden), which have a 5 mm projected width at isocenter.

### 2.1 Patient cohort

Seventeen patients with multiple brain metastases were included in this study, for a total of 78 lesions treated with single‐fraction cranial SRS. Patients individually presented with 2−10 metastases ranging in lesion volume from 0.010 cc to 4.980 cc. Prescription doses were assigned by lesion size, with approval from the physician following Table 1.

**TABLE 1:** SRS prescription doses assigned by PTV size.

Lesion size (longest PTV dimension)	Prescription dose (98.0% of volume)
<1.0 cm	24 Gy
1.0 to <2.0 cm	22 Gy
2.0 cm to 3.0 cm	20 Gy

Abbreviation: PTV: planning target volume.

Treatment plan isocenter was placed by the Brainlab system such that it was located at the approximate geometric center of the treated field. Based on the distance between the isocenter and each target, treatment margins were added to each gross tumor volume (GTV) to create planning target volumes (PTV). As routine quality assurance shows that our LINAC faithfully meets a 1.0 mm tolerance at isocenter and a 1.5 mm tolerance for targets greater than 5 cm off‐axis, PTV margins were 1.0 mm at minimum, but were increased to 1.5 mm for targets more than 5 cm away from treatment isocenter. The additional margin accounted for magnification of rotational uncertainties in patient setup and collimator position for off‐axis targets. Margins were additionally increased as needed for lesions smaller than 4 mm × 4 mm × 4 mm following discrepancies between measured and planned distributions found in commissioning.

Case‐by‐case margins, prescriptions, and range of separations between lesions and isocenter for the patient cohort are listed in Table 2.

**TABLE 2:** Summary of patient cases and prescription doses used for this study.

Case	Treated mets	Minimum GTV vol. [cc]	Maximum GTV vol. [cc]	Minimum distance to iso [cm]	Maximum distance to iso [cm]	PTV prescription doses (margin)	
1	2	0.015	0.033	0.87	0.87	PTV1 (2.0 mm): 22 Gy,	PTV2 (2.0 mm): 22 Gy
2	2	0.311	0.675	1.72	1.74	PTV1 (1.0 mm): 22 Gy	PTV2 (1.0 mm): 22 Gy
3	2	0.084	0.387	4.67	4.67	PTV1 (1.0 mm): 22 Gy	PTV2 (1.0 mm): 20 Gy
4	2	0.294	0.757	4.27	4.28	PTV1 (1.0 mm): 22 Gy	PTV2 (1.0 mm): 22 Gy
5	2	0.016	0.089	0.52	0.52	PTV1 (1.5 mm): 24 Gy	PTV2 (1.0 mm): 24 Gy
6	2	0.482	0.570	1.92	1.94	PTV1 (1.0 mm): 20 Gy	PTV2 (1.0 mm): 24 Gy
7	2	0.390	0.508	2.74	2.76	PTV1 (1.0 mm): 22 Gy	PTV2 (1.0 mm): 22 Gy
8	2	0.065	0.106	2.08	2.08	PTV1 (1.0 mm): 24 Gy	PTV2 (1.0 mm): 24 Gy
9	4	0.094	0.418	2.27	7.83	PTV1 (1.5 mm): 22 Gy PTV2 (1.5 mm): 22 Gy	PTV3 (1.5 mm): 22 Gy PTV4 (1.5 mm): 24 Gy
10	4	0.053	1.615	2.61	7.35	PTV1 (1.5 mm): 24 Gy PTV2 (1.0 mm): 22 Gy	PTV3 (1.0 mm): 24 Gy PTV4 (1.5 mm): 22 Gy
11	6	0.049	0.747	4.34	7.35	PTV1 (1.5 mm): 22 Gy PTV2 (1.5 mm): 22 Gy PTV3 (1.5 mm): 22 Gy	PTV4 (1.5 mm): 22 Gy PTV5 (1.0 mm): 22 Gy PTV6 (1.5 mm): 24 Gy
12	6	0.010	0.237	1.95	7.01	PTV1 (1.5 mm): 24 Gy PTV2 (1.5 mm): 24 Gy PTV3 (1.5 mm): 22 Gy	PTV4 (1.5 mm): 24 Gy PTV5 (1.5 mm): 24 Gy PTV6 (1.5 mm): 24 Gy
13	7	0.022	1.655	4.60	6.86	PTV1 (1.5 mm): 22 Gy PTV2 (1.5 mm): 22 Gy PTV3 (1.5 mm): 22 Gy PTV4 (1.5 mm): 20 Gy	PTV5 (1.5 mm): 24 Gy PTV6 (1.0 mm): 22 Gy PTV7 (1.5 mm): 22 Gy
14	8	0.023	0.355	3.08	8.78	PTV1 (1.5 mm): 24 Gy PTV2 (1.5 mm): 24 Gy PTV3 (1.5 mm): 20 Gy PTV4 (2.0 mm): 20 Gy	PTV5 (2.0 mm): 24 Gy PTV6 (1.0 mm): 20 Gy PTV7 (1.5 mm): 20 Gy PTV8 (1.5 mm): 20 Gy
15	8	0.011	0.067	3.06	8.59	PTV1 (1.0 mm): 24 Gy PTV2 (1.0 mm): 24 Gy PTV3 (1.0 mm): 24 Gy PTV4 (1.0 mm): 24 Gy	PTV5 (1.0 mm): 24 Gy PTV6 (1.0 mm): 24 Gy PTV7 (1.5 mm): 24 Gy PTV8 (1.5 mm): 24 Gy
16	9	0.032	4.980	1.73	7.70	PTV1 (1.5 mm): 20 Gy PTV2 (1.5 mm): 20 Gy PTV3 (1.5 mm): 22 Gy PTV4 (1.0 mm): 22 Gy PTV5 (1.0 mm): 22 Gy	PTV6 (1.5 mm): 22 Gy PTV7 (1.0 mm): 22 Gy PTV8 (1.5 mm): 22 Gy PTV9 (1.5 mm): 22 Gy
17	10	0.028	0.556	3.53	7.83	PTV1 (1.5 mm): 24 Gy PTV2 (1.0 mm): 24 Gy PTV3 (1.5 mm): 24 Gy PTV4 (1.5 mm): 24 Gy PTV5 (1.5 mm): 22 Gy	PTV6 (1.0 mm): 22 Gy PTV7 (1.0 mm): 22 Gy PTV8 (1.5 mm): 24 Gy PTV9 (1.5 mm): 24 Gy PTV10 (1.5 mm): 24 Gy

Abbreviations: GTV: gross tumor volume, Iso: treatment isocenter, PTV: planning target volume.

### 2.2 Treatment planning

**FIGURE 1:**
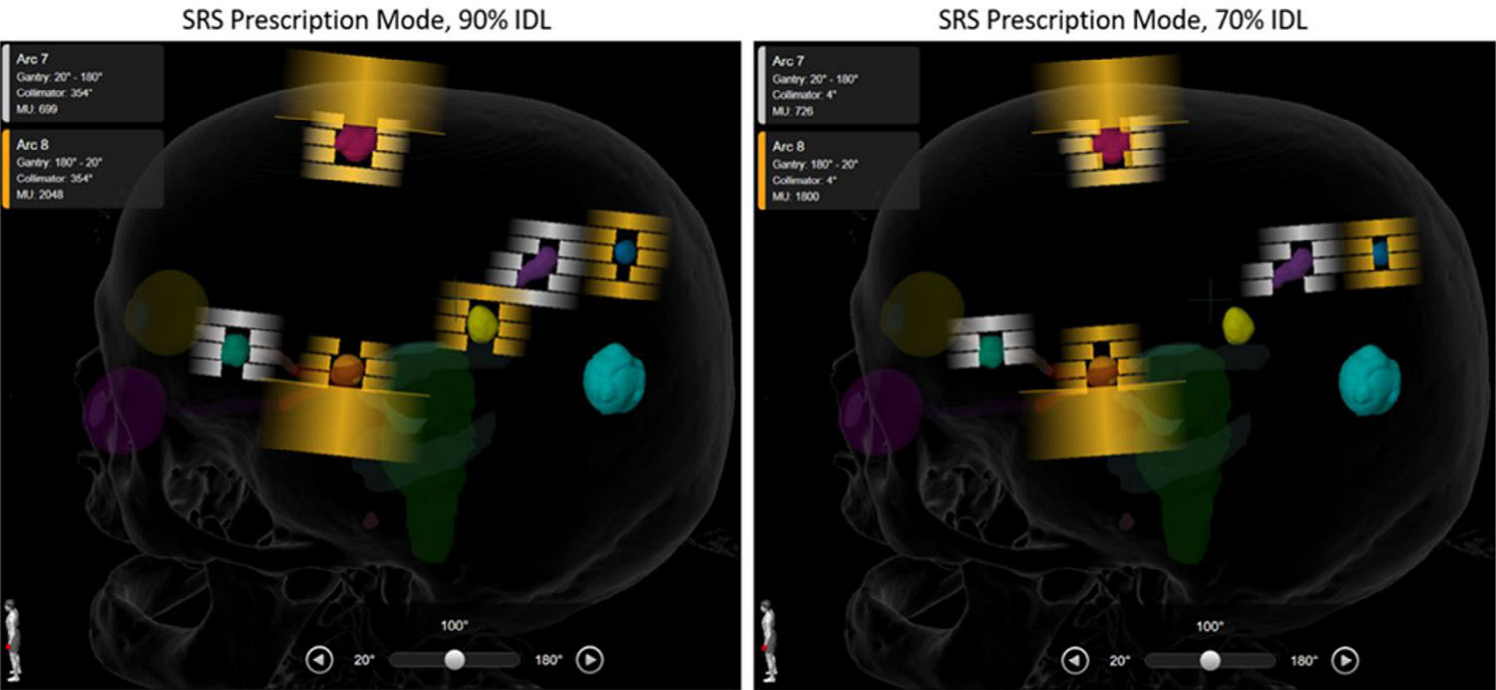
Side‐by‐side comparison of optimization using the 90% prescription isodose line (IDL) and 70% IDL. At the lower IDL, collimator leaves close the aperture and partially obscure the target in the beam's eye view, creating the “negative margin.”

Dynamic conformal arc plans were optimized in Brainlab Multi‐Met Elements v3.0 (Brainlab, Munich, Germany) using the built‐in “SRS Prescription” mode, which allows users to specify a desired prescription IDL. When selecting a lower prescription IDL, the MLC aperture shrinks, closing the field and creating “negative margins” in the beam's eye view, where the target is partially obscured by MLCs at low prescription IDL (Figure 1).

Clinically treated plans used the 80% prescription IDL as standard and were further optimized by users to meet clinical goals and facilitate treatment delivery as needed. All plans were approved by a physician for treatment and delivered. Retroactive optimizations using 70%−90% prescription IDL in 5% increments were then performed. These re‐optimizations used the same treatment parameters including avoidance structures, couch angles, and gantry arcs as the clinically treated plans, which maintained the clinical relevance of each plan and isolated the IDL as the optimization parameter of interest. The resulting dosimetric parameters for each IDL optimization were recorded for analysis.

### 2.3 Dosimetric parameters

#### 2.3.1 Volume of tissue, V10, V12, and V15

V12 was defined here as the volume of brain tissue receiving 12 Gy or more, minus the GTV (Equation 1).

(1) $$V12=V_{brain,12Gy}-GTV$$

The V10 and V15 were defined accordingly at the 10 and 15 Gy dose levels, respectively.

#### 2.3.2 Monitor units

The treatment MU were taken as the total treatment MU calculated by the TPS for each optimization. As the number of gantry arcs and their lengths are consistent between optimizations, the treatment MU is a direct indicator of a difference in treatment delivery time.

#### 2.3.3 Conformity index

Conformity between optimizations was compared using the inverse Paddick Conformity Index (CI) calculated in the TPS (Equation 2).

(2) $$CI=\frac{PTV\cdot PIV}{{\left(TTV\right)}^{2}}$$

*TTV* is the total treated volume (the volume of target receiving the prescription dose), *PTV* is the planning target volume, and *PIV* is the prescription isodose volume (the volume of tissue receiving the prescription dose). For the total plan comparison, we used the volume‐averaged CI calculated in Brainlab Elements TPS. As defined, a conformity index near unity indicates better alignment between the prescription isodose volume and the target volume.

### 2.4 Data analysis

V10, V12, V15, MU, and CI data were presented as the percent difference (*%Δ)* to the 80% prescription IDL plan. Normalizing to a selected prescription IDL isolated the effects of the prescription IDL on our dosimetric parameters for comparison between cases.

Statistical analysis was carried out using analysis of variance (ANOVA) in Origin (Originlab, Northampton, MA). Differences in parameters between optimizations were determined to be statistically significant at the confidence interval *p* < 0.05. For *p* > 0.05, differences were considered not statistically significant (NS).

## 3 RESULTS

A summary of the study results is presented in Table 3.

**TABLE 3:** Summary of results presented as average percent differences to the 80% IDL optimization.

		Average % difference to 80% IDL, SD			
	Metric	70% IDL	75% IDL	85% IDL	90% IDL
2 metastases (*N* = 8)	MU	17.0 *± 3.2*	7.7 *± 2.4*	−6.8 *± 5.2*	−13.6 *± 5.6*
	CI	1.0 *± 5.7*	0.3 *± 6.9*	0.0 *± 8.9*	2.0 *± 10.8*
	V10	−7.7 *± 5.6*	−4.9 *± 5.1*	11.6 *± 6.2*	36.6 *± 13.4*
	V12	−8.4 *± 5.7*	−4.9 *± 5.1*	12.8 *± 6.5*	39.5 *± 15.5*
	V15	−8.9 *± 5.5*	−5.0 *± 5.0*	14.5 *± 6.7*	43.8 *± 18.2*
4‐6 metastases (*N* = 4)	MU	15.4 *± 6.1*	10.1 *± 3.4*	−7.2 *± 11.9*	−7.3 *± 8.6*
	CI	2.1 *± 1.5*	0.8 *± 2.2*	0.1 *± 3.6*	6.8 *± 1.2*
	V10	−5.4 *± 5.5*	−5.6 *± 2.5*	9.9 *± 7.4*	32.8 *± 3.7*
	V12	−4.6 *± 5.2*	−4.7 *± 2.9*	12.5 *± 7.9*	36.9 *± 5.2*
	V15	−5.0 *± 6.2*	−4.9 *± 4.0*	14.0 *± 7.9*	39.8 *± 6.2*
7‐10 metastases (*N* = 5)	MU	12.2 *± 13.6*	3.0 *± 11.7*	−6.7 *± 4.3*	−7.2 *± 4.4*
	CI	5.6 *± 9.4*	3.5 *± 6.2*	3.2 *± 3.7*	8.6 *± 5.6*
	V10	−2.3 *± 2.7*	−2.0 *± 5.5*	14.1 *± 3.0*	30.2 *± 13.2*
	V12	−2.5 *± 3.4*	−1.1 *± 5.3*	15.1 *± 2.7*	33.9 *± 11.0*
	V15	−3.0 *± 3.8*	−0.9 *± 5.8*	17.1 *± 2.9*	38.5 *± 7.9*

Abbreviations: CI, conformity index; IDL, prescription isodose line; MU, monitor units; SD, standard deviation.

### 3.1 Effect of prescription IDL on V10, V12, and V15

#### 3.1.1 Two treated brain metastases

**FIGURE 2:**
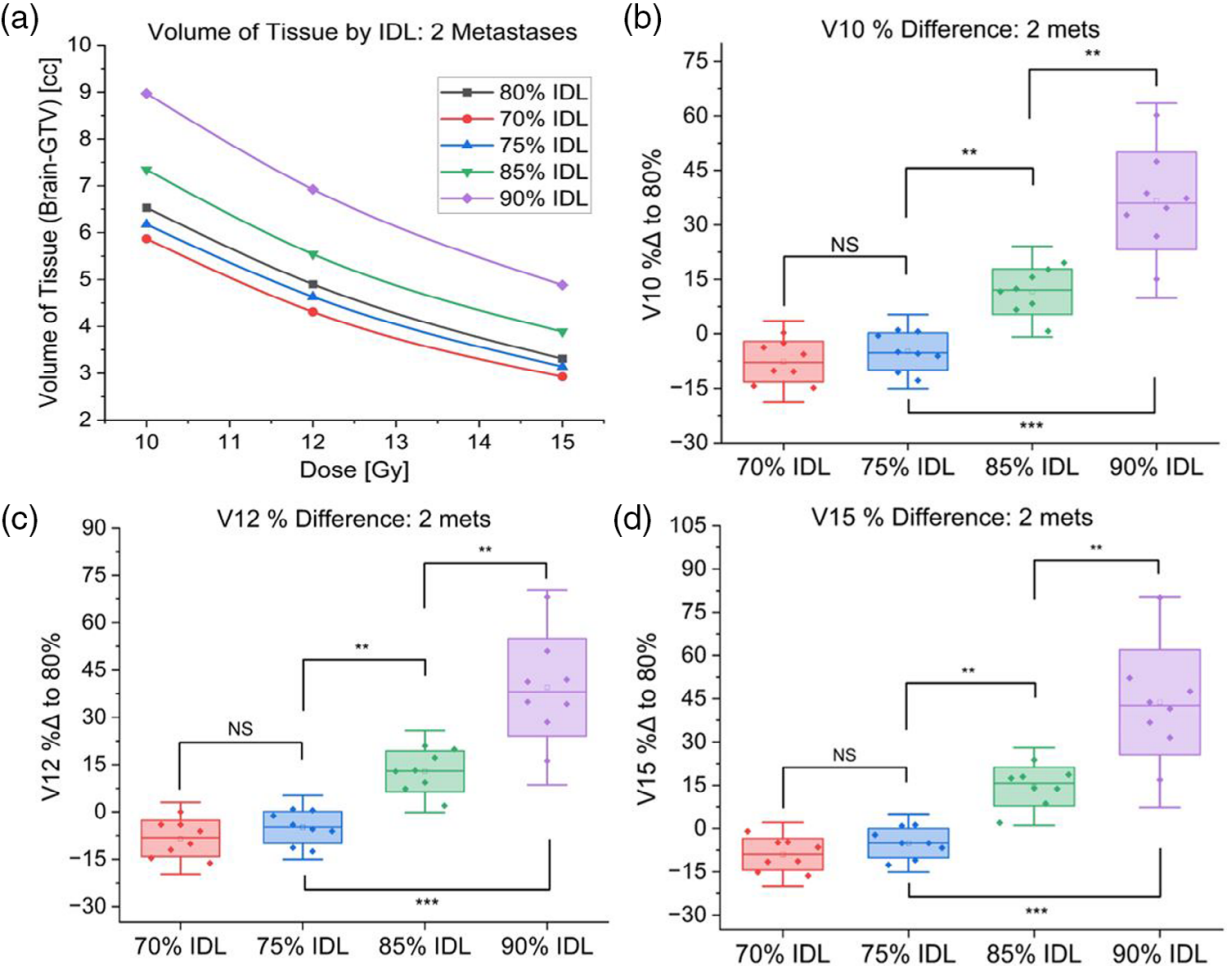
Results of 70−90% prescription IDL optimizations for plans treating two metastases. (a) Volume of brain tissue receiving specified dose for each IDL optimization for a representative case treating two metastases. (b) Comparison of V10 for cases treating two metastases optimized at 70−90% prescription IDLs. (c) Comparison of V12 for cases treating two metastases optimized at 70−90% prescription IDLs. (d) Comparison of V15 for cases treating two metastases optimized at 70−90% prescription IDLs (NS: Not statistically significant, *p < 0.05, **p < 0.01, ***p < 0.001).

As shown in Table 3, relative to the 80% prescription IDL, optimization at the 70% IDL yielded a 7.7% ± 5.6% reduction in V10, 8.4% ± 5.7% reduction in V12, and an 8.9% ± 5.5% reduction in V15. Conversely, optimization at the 90% IDL resulted in a 36.6% ± 13.4% increase in V10, 39.5% ± 15.5% increase in V12, and 43.8% ± 18.2% increase in V15 (Table 3, Figure 2).

**FIGURE 3:**
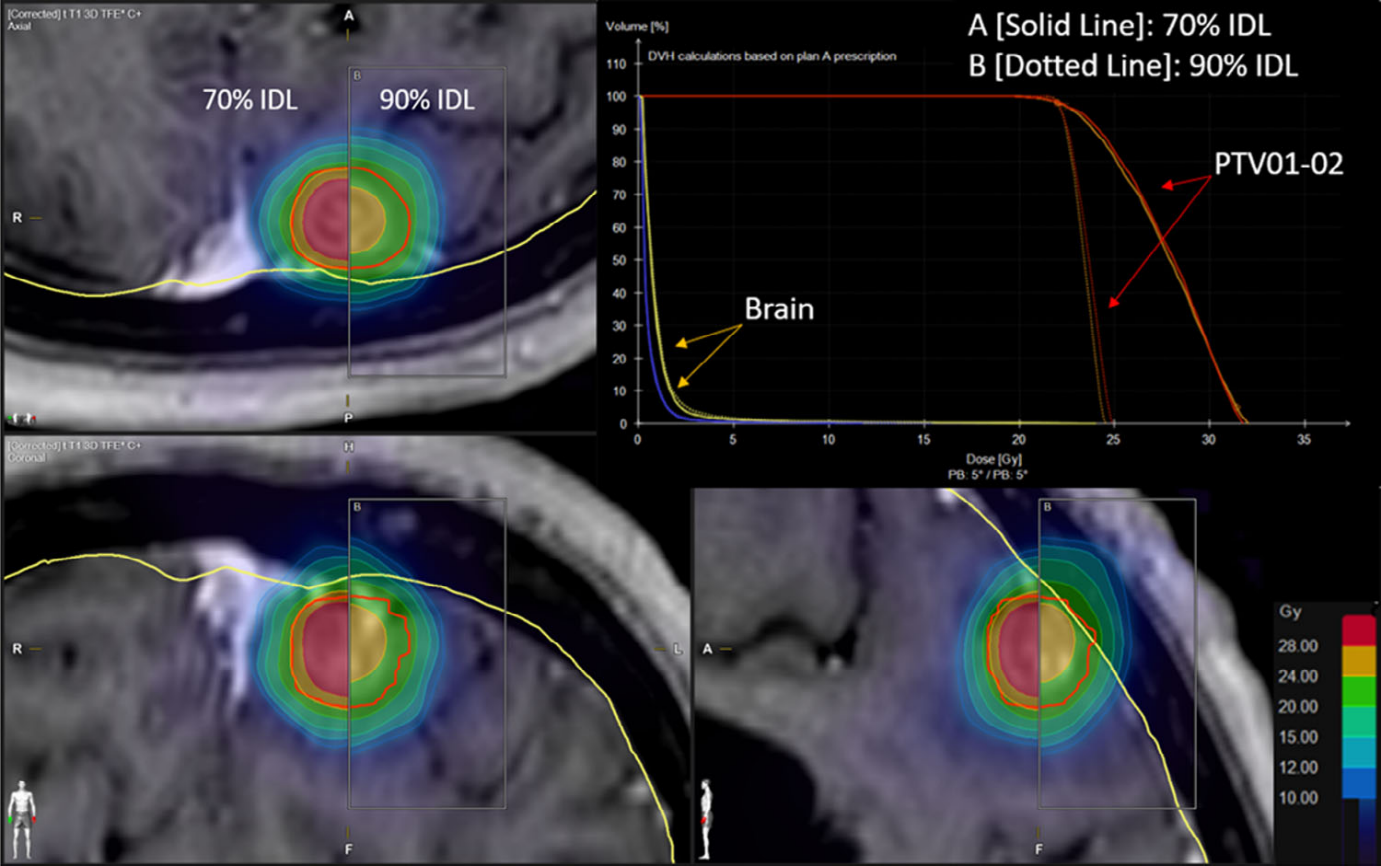
Comparison of dose distributions and dose‐volume histogram (DVH) for 70% (solid lines) and 90% (dotted lines) prescription isodose line optimizations for a plan treating two metastases.

Overall, relative to the 80% IDL, optimization at 70% versus 75% IDLs showed no statistically significant differences in V10, V12, or V15 (Figure 2). However, the 85% and 90% IDLs had significantly higher V10, V12, and V15 (*p* < 0.01). Particularly, the 90% IDL optimization irradiated a dramatically greater tissue volume (*p* < 0.01) than any other prescription IDL when treating two metastases (Figure 3).

#### 3.1.2 Four to six treated brain metastases

**FIGURE 4:**
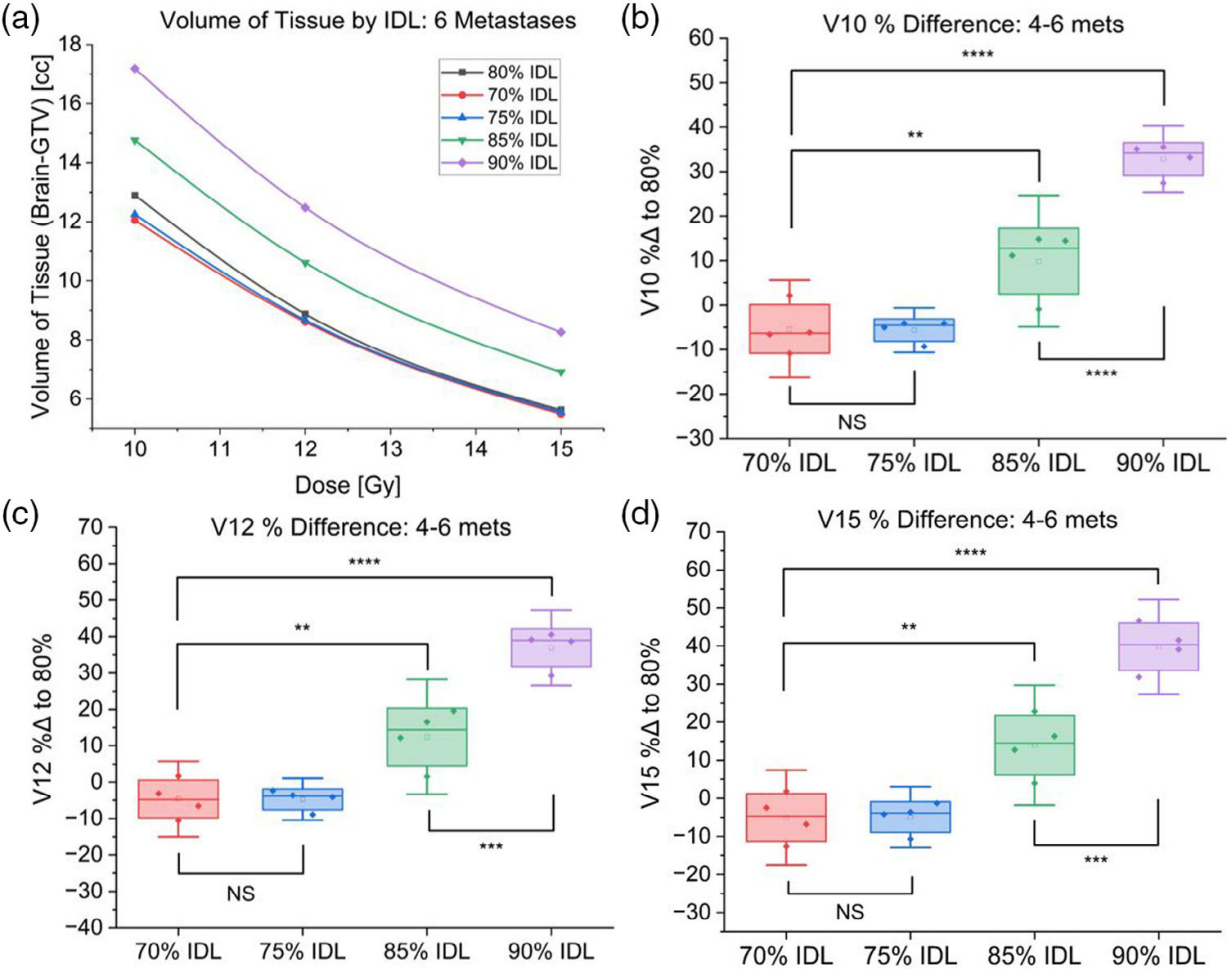
Results of 70−90% prescription IDL optimizations for plans treating four to six metastases. (a) Volume of brain tissue receiving specified dose for each IDL optimization for a representative case treating six metastases. (b) Comparison of V10 for cases treating four to six metastases optimized at 70−90% prescription IDLs. (c) Comparison of V12 for cases treating four to six metastases optimized at 70−90% prescription IDLs. (d) Comparison of V15 for cases treating four to six metastases optimized at 70−90% prescription IDLs (NS: Not statistically significant, *p < 0.05, **p < 0.01, ***p < 0.001)​.

For four to six treated metastases, relative to the 80% IDL, optimization at the 70% IDL resulted in a 5.4% ± 5.5% decrease in V10, a 4.6% ± 5.2% decrease in V12, and a 5.0% ± 6.2% decrease in V15. The 90% IDL again irradiated significantly more tissue (*p* < 0.01), with a 32.8% ± 3.7% increase in V10, 36.9% ± 5.2% increase in V12, and a 39.8% ± 6.2% increase in V15 (Table 3, Figure 4).

**FIGURE 5:**
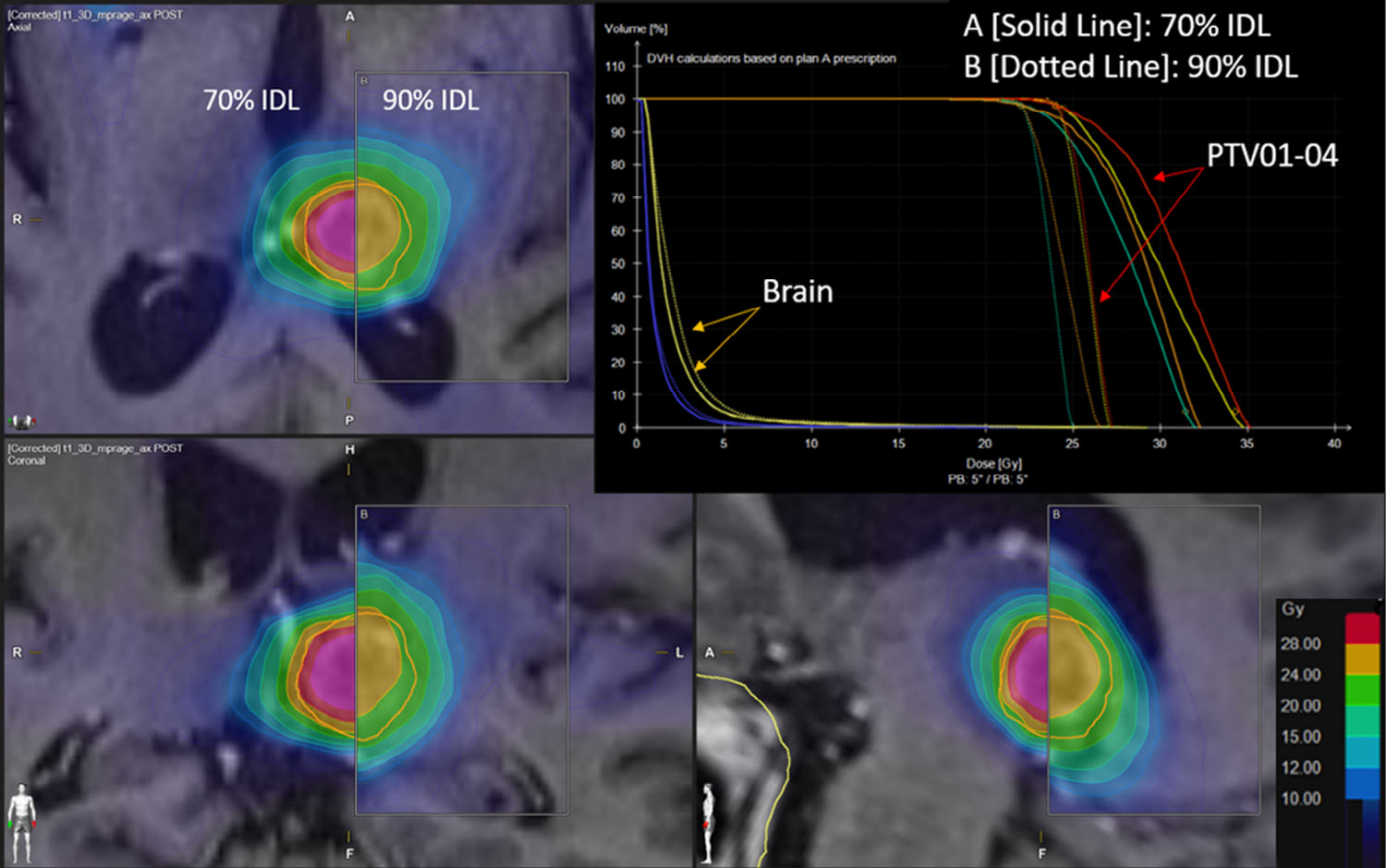
Comparison of dose distributions and dose‐volume histogram (DVH) for 70% (solid lines) and 90% (dotted lines) prescription isodose line optimizations for a plan treating four metastases.​

There was again no statistically significant difference in V10, V12, or V15 for optimizations at the 70% and 75% IDLs (Figure 4), however, as seen with two metastases, there was a dramatic improvement in tissue sparing when optimizing at IDLs below 90% (*p* < 0.01) (Figures 4 and 5).

#### 3.1.3 Seven to ten treated brain metastases

**FIGURE 6:**
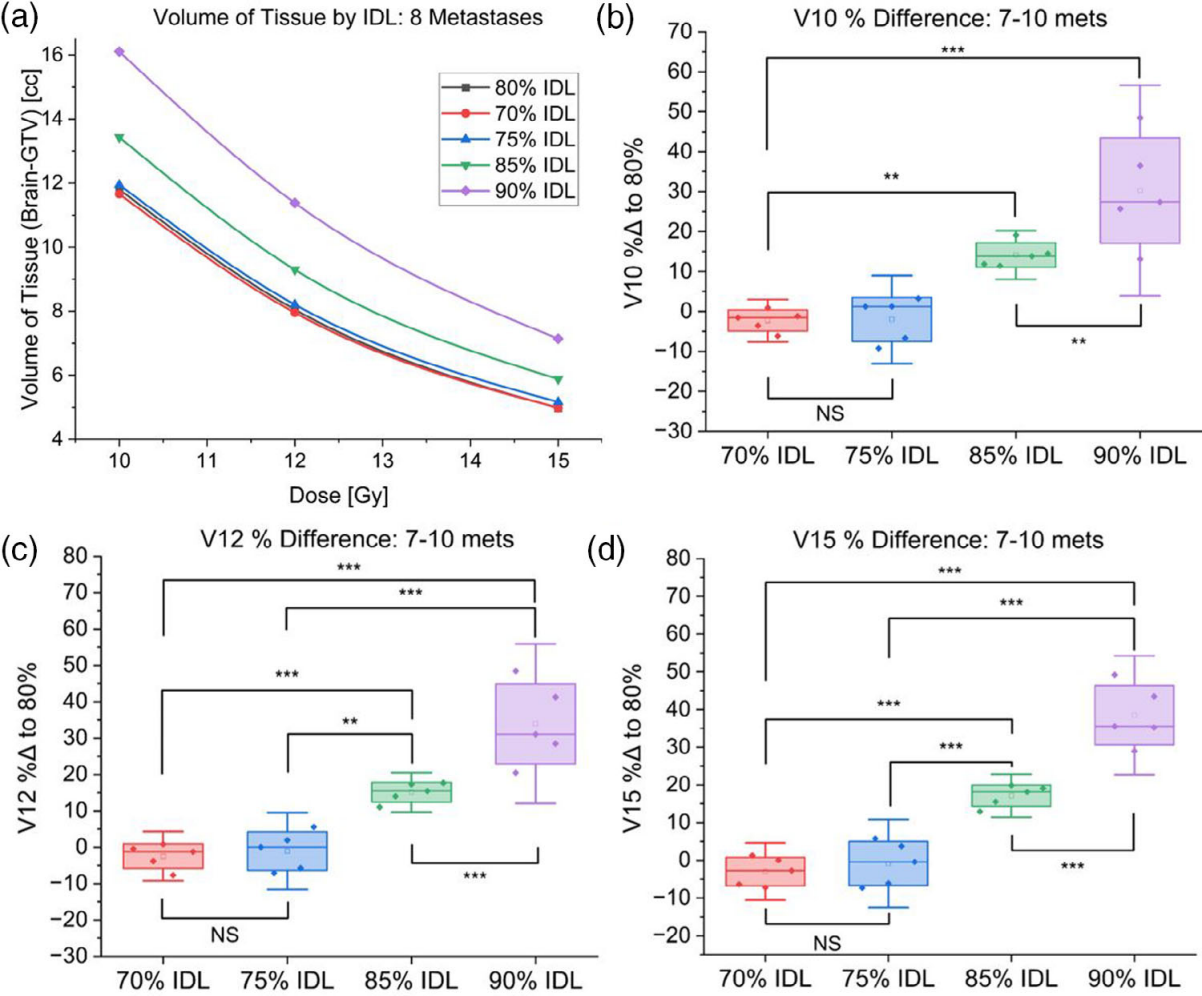
Results of 70−90% prescription IDL optimizations for plans treating 7−10 metastases. (a) Volume of brain tissue receiving specified dose for each IDL optimization for a representative case treating eight metastases. (b) Comparison of V10 for cases treating 7−10 metastases optimized at 70−90% prescription IDLs. (c) Comparison of V12 for cases treating 7−10 metastases optimized at 70−90% prescription IDLs. (d) Comparison of V15 for cases treating 7−10 metastases optimized at 70−90% prescription IDLs (NS: Not statistically significant, *p < 0.05, **p < 0.01, ***p < 0.001)​.

Finally, for 7−10 metastases, relative to the 80% IDL, prescribing to the 70% IDL lowered V10 by 2.3% ± 2.7%, V12 by 2.5% ± 3.4%, and V15 by 3.0% ± 3.8%. The 90% IDL accordingly increased V10 by 30.2% ± 13.2%, V12 by 33.9% ± 11.0%, and V15 by 38.5% ± 7.9% (Table 3, Figure 6).

**FIGURE 7:**
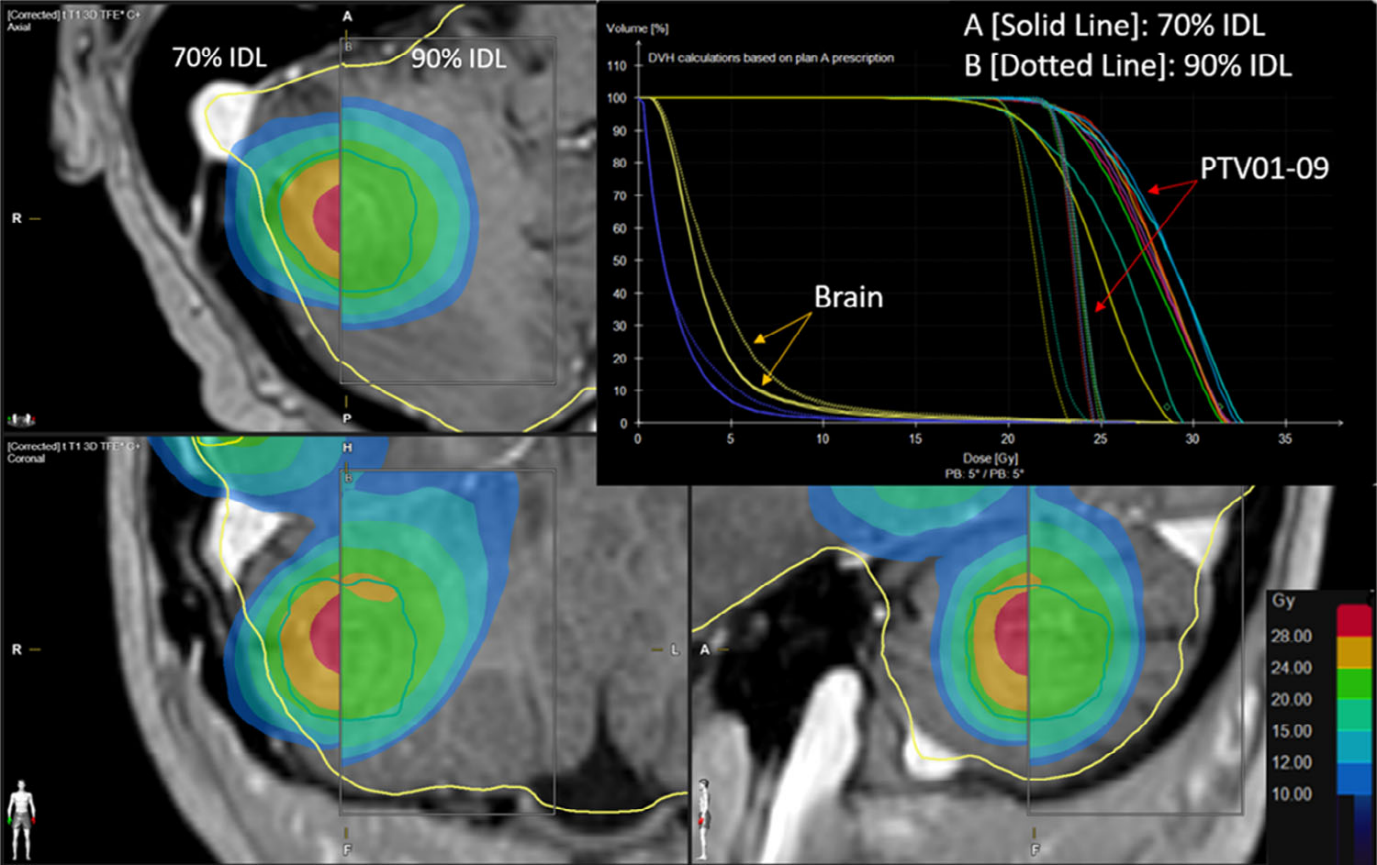
Comparison of dose distributions and dose‐volume histogram (DVH) for 70% (solid lines) and 90% (dotted lines) prescription isodose line optimizations for a plan treating six metastases.

There is again dramatic improvement in tissue sparing for IDLs below 90% (*p* < 0.01), and no statistically significant differences in tissue sparing at IDLs below 80% (Figure 6). Additionally, the differences in tissue sparing at dose levels below 10 Gy are noticeably greater for cases treating increasingly many targets (Figure 7).

### 3.2 Effect of prescription IDL on treatment MU

**FIGURE 8:**
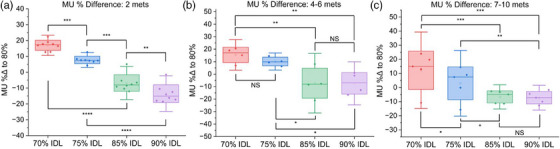
Comparison of treatment monitor units (MU) for plans optimized at the 70−90% IDLs. (a) Results for plans treating two metastases. (b) Results for plans treating four to six metastases. (b) Results for plans treating 7−10 metastases (NS: Not statistically significant, *p < 0.05, **p < 0.01, ***p < 0.001)​.

For 2 treated metastases, plans using the 70% IDL called for 17.0% ± 3.2% greater treatment MU, whereas at the 90% IDL, treatment MU decreased by 13.6% ± 5.6%, relative to the 80% IDL optimization (Figure 8a).

For four to six treated metastases, the prescription IDL had a smaller effect on treatment MU. Relative to the 80% IDL, optimization at the 70% IDL increased treatment MU by 15.4% ± 6.1%, and the 90% IDL decreased MU by 7.3% ± 8.6%. There was thus a significant reduction in MU when using IDLs above 75% (*p* < 0.01), but when optimizing at the 80% IDL, in contrast to the cases treating two metastases, there was little MU reduction when moving to higher prescription IDLs for cases treating four to six metastases (Figure 8b). In these cases, there was a statistically significant increase in MU when using prescription IDLs below 80% compared to IDLs greater than 80% (*p* < 0.05), however, there was no statistically significant difference in treatment MU when moving from 90% to 85%, or 75% to 70% IDLs.

For 7−10 metastases, 70% IDL optimization resulted in a 12.2% ± 13.6% increase in treatment MU, and the 90% IDL lowered MU by 7.2% ± 4.4% relative to the 80% IDL. There was no statistically significant increase in MU when optimizing at the 85% IDL compared to the 90% IDL, however, optimization at the 70% prescription IDL required greater treatment MU (*p* < 0.01) (Figure 8c).

### 3.3 Effect of prescription IDL on conformity index

**FIGURE 9:**
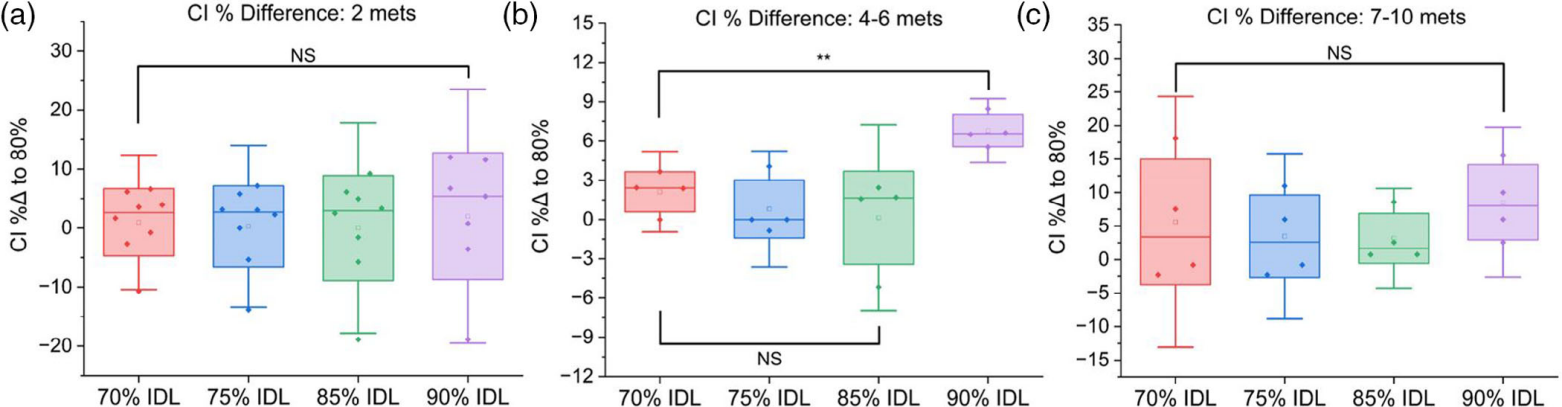
Comparison of conformity index (CI) for plans optimized at the 70−90% IDLs. (a) Results for plans treating two metastases. (b) Results for plans treating four to six metastases. (b) Results for plans treating 7−10 metastases (NS: Not statistically significant, *p < 0.05, **p < 0.01, ***p < 0.001).

In contrast to the effects seen in tissue sparing and MU, the overall effect of the prescription IDL on the conformity index was not statistically significant, except when treating four to six metastases and comparing the 90% IDL conformity index to the 70% IDL CI (Figure 9).

## 4 DISCUSSION

In modern radiation oncology clinics, physicians, physicists, and dosimetrists must balance several key factors to produce an optimal treatment plan. For cranial SRS, the fundamental issues at hand include irradiation of healthy tissue, treatment time, and target coverage. Since SRS uses single fraction high biological‐equivalent doses, achieving local control while minimizing the probability of tissue necrosis remains the key cost‐benefit for cranial radiosurgery.

In this study, we have observed and quantified the effect of the prescription IDL on tissue sparing (V10, V12, V15), treatment MU, and conformity index for plans treating 2−10 brain metastases in the novel Brianlab Elements v3.0 TPS using DCA and a single treatment isocenter.

For tissue sparing, our results showed a consistent effect irrespective of the number of treated metastases. Moving from the 90% IDL optimization to the 85% IDL significantly decreased V10, V12, and V15 for 2−10 treated metastases (*p* < 0.01). Furthermore, the 75% IDL spared significantly more tissue than the 85% optimization (*p* < 0.01); however, optimization at the 70% IDL showed no significant improvement in tissue sparing over the 75% IDL. Therefore, we observed a significant benefit to using prescription IDL below 85%. However, progressively lower prescription IDLs also yielded diminishing returns for tissue sparing, as demonstrated for representative cases in Figures 2, 4, and 6a.

Treatment MU and CI followed a more case‐dependent trend. For two metastases, there was a clear and significant increase in MU at lower prescription IDL (*p* < 0.01), which correlated with a longer treatment time. Though less pronounced, the overall effect was similar for 4−10 treated metastases. The conformity index, however, exhibited a large spread in data due to strong case dependence, and therefore showed no consistent trend for each prescription IDL optimization.

In addition to the effects on tissue sparing, MU, and CI, users should also consider the introduction of dosimetric hot spots when selecting prescription IDL. A lower prescription IDL increases the maximum dose in the target, which places additional demands on patient immobilization and machine geometric accuracy.

For our plans treating up to 10 metastases, diminishing returns for tissue sparing at IDLs below 80% paired with increasing treatment MU and dosimetric hot spot made optimization at lower IDLs less favorable.

Our results confirm that optimization at the 80% IDL achieves the best balance of V12, treatment MU, and maximum dose irrespective of the number of treated metastases, validating the continued use of the 80% prescription IDL as our clinical standard, which allows us to reduce overall planning time by fixing the prescription IDL at the preferred configuration. Although standardized in our clinic, users still have the option to adjust the prescription IDL in situations where the plan may benefit from other optimizations. A common example is treatment of post‐surgical cavities, for which we typically select the 90% IDL in favor of the lower hot spot and shallower dose falloff surrounding the cavity.

Though in this study, the TPS was allowed to change MLC apertures, MU, and collimator angles across percent IDL optimizations, the same gantry angles, arcs, and couch angles were used for each optimization. Though this method allowed us to isolate and observe the effect of the prescription IDL on our dosimetric parameters, a key limitation of this study was the lack of bespoke manual intervention for each prescription IDL optimization. Future work may therefore involve manual user input in reoptimizing plans at each prescription IDL.

## 5 CONCLUSION

By observing the effect of the prescription IDL on various plan metrics, we have shown that standardizing the prescription IDL is a viable strategy for reducing planning time without sacrificing plan quality. Similar analysis may be used by other clinics developing their own protocols to find the point at which improvement in tissue sparing, treatment MU, and hot spot are best balanced. Additionally, the results presented herein set the stage for potential future comparisons between linear accelerator‐based SRS and other SRS platforms.

## AUTHOR CONTRIBUTIONS

All authors have made substantial contributions to the design and implementation of the presented work, including acquisition, analysis, and interpretation of data, manuscript drafting and revision.

## CONFLICT OF INTEREST STATEMENT

The authors declare no conflicts of interest.
